# Narcissistic symptoms among Iranian outer-city bus drivers

**DOI:** 10.5249/jivr.v12i3.1517

**Published:** 2020-08

**Authors:** Leila Tabrizi, Ahmad Karbalaee, Sara Pashang

**Affiliations:** ^*a*^ Department of Psychology, Faculty of Psychology, Karaj Branch, Islamic Azad University, Alborz, Iran.

**Keywords:** Personality, Psychology, Road traffic injuries, High risk drivers

## Abstract

**Background:**

Although very few studies have investigated the association of narcissistic symptoms and aggressive driving, very little is known about association of narcissism and serious traffic outcomes such as crash and serious violation of traffic laws. The aim of this study was to determine whether there is an association between the narcissistic symptoms of professional bus drivers and high risk driving records or crash.

**Methods:**

A total of 200 outer-city bus drivers were enrolled in 2018 from Tehran origin of trips. The narcissistic symptoms of drivers were assessed using the Narcissistic Personality Inventory-16 (NPI-16). The traffic police databases were searched for records of crashes or recorded negative traffic scores during a 3-year period prior to time of interviews. Data were analyzed using Stata 14 statistical software package.

**Results:**

Mean age of the participants was 44.4 years with a standard deviation of 9.3 years. Fourteen drivers (7%) had a crash history over the past three years. Mean normalized narcissism score was 22.3 among those without a crash history over the past three years versus 18.8 among those with a crash history without statistical significance. Forty-four drivers (22%) had a negative traffic scoring record due to high risk traffic violations registered in police database over the past three years. Mean normalized narcissism score was 22 among those without negative score record over the past three years versus 22.3 among those with a negative score history. However, the difference was not found to be statistically significant.

**Conclusions:**

The findings of present study does not support an association between crash risk or being a recorded high risk driver and narcissism levels. However, considering the complex risk profile of road traffic crashes, much larger studies are needed to rule it out.

## Introduction

Road traffic injuries are a global health challenge especially in low- and middle income countries (LMICs). Approximately 1.35 million people die each year as a result of road traffic crashes. Ninety-three percent of the global fatalities on the roads occur in LMICs, while these countries own about 60% of the world's vehicles.^1^ Road traffic injuries are also a major health problem in Iran. The total cost of RTIs has been reported to reach up to 7.2 billion US Dollars yearly comprising 2.19% of Iran’s Gross Domestic Production^2^ Traffic psychology is a main field of road safety research. Driver personality has been found to be a significant predictor of driving behavior.^3^ In field of personality traits, the narcissism has a long history in social psychology and clinical psychology. Narcissism is a clinical disorder of personality that is based on a set of diagnostic criteria, whether or not they are diagnosed. This is while social psychologists generally have a dimensional view of narcissism. There is no definite boundary in the scope of narcissism that distinguishes between normal and narcissistic. People with narcissistic personality tend to be superior, more specific, and more unique than others and are extremely sensitive to criticism, thus having difficulty in important areas of life such as work, education, and interpersonal relationships. Narcissistic personality presents with signs of grandiosity, mental occupation, fantasies of success, power, beauty talent, the belief that the individual is exceptional, feeling all-powerful, exploiting interpersonal relationships and lack of empathy which in turn gives rise to extreme praise and egoism. On the other hand, research has made it clear that narcissism is associated with adaptation.^4^ Based on threatened egotism theory, it has been argued that narcissism could lead to aggressive behavior such that subjects having high unstable self-esteem may respond defensively with anger or aggressive behavior each time they perceive a threat to their favorable self-appraisals.^5,6^ This situation could be assumed also to occur in transportation environment when drivers are subject to being offended or threatened by traffic behaviors of other drivers or other people involved in transportation environment. The resulting potential aggressive impulses may lead to aggressive driving that in turn could increase the risk of unwanted traffic outcomes such as violation of traffic laws and crashes. Although very few studies have investigated the association of narcissistic symptoms and aggressive driving,^7,8^ very little is known about association of narcissism and serious traffic outcomes such as crash and serious violation of traffic laws. The aim of current exploratory study was to determine whether there is an association between the narcissistic symptoms of professional bus drivers and high risk driving records or crashes.

## Methods 

The cross-sectional study was conducted in 2018 in Tehran, Iran. The study population included bus drivers registered as professional outer-city bus driver by Iranian Road Administration and Transportation Organization. A total of 200 drivers available out of the total 5000 registered drivers were selected through consecutive convenient sampling. They were interviewed by a trained psychologist based on the study protocol. Questionnaires were delivered to bus drivers who stated their willingness to participate in the study under the conditions of the ethical requirements of the study and the standards of conducting the research. They were asked to answer both the few basic questions such as age, gender, education and driving experience (in years) as well as the questions on narcissism assessment inventory. They were told to carefully and honestly answer all the questions, but not to spend too much time on any specific item. Narcissistic Personality Inventory-16 (NPI-16) was used as narcissism assessment tool. The NPI-16 is a short measure of subclinical narcissism, developed by Ames in 2006, consists of 16 pairs of sentences designed to measure narcissistic personality traits. One statement in each of the 16 pairs of items is considered as indicative of narcissistic symptoms. For each person the proportion of responses consistent with narcissism was computed and used as narcissistic score. It is used as an alternative scale for narcissism when situations do not allow the use of longer inventories. It has shown to have acceptable face validity, internal validity, discriminant validity, and predictive validity.9 The Persian version of NPI-16 has also been found to be valid and reliable.^10^ Moreover, the traffic police databases were searched for records of crashes or recorded negative traffic scores during a 3-year period prior to the time of interviews.

Data were analyzed using Stata version 14 statistical software package. Descriptive and analytical methods were applied. Normality of numeric scales were checked using quintile normal plot assessment. The narcissistic level for drivers were calculated as a total score normalized to a range of 0-100 min/max levels. The association of narcissistic level with study outcomes was assessed using independent t-test. A p-value<0.05 was considered as statistical significance margin. 

The study was approved by high degree research committee board at Islamic Azad University of Iran and according to the reviewed codes of ethics. The drivers were assured that their answers to the questions would be treated in confidence. 

## Results

**Figure 1:**
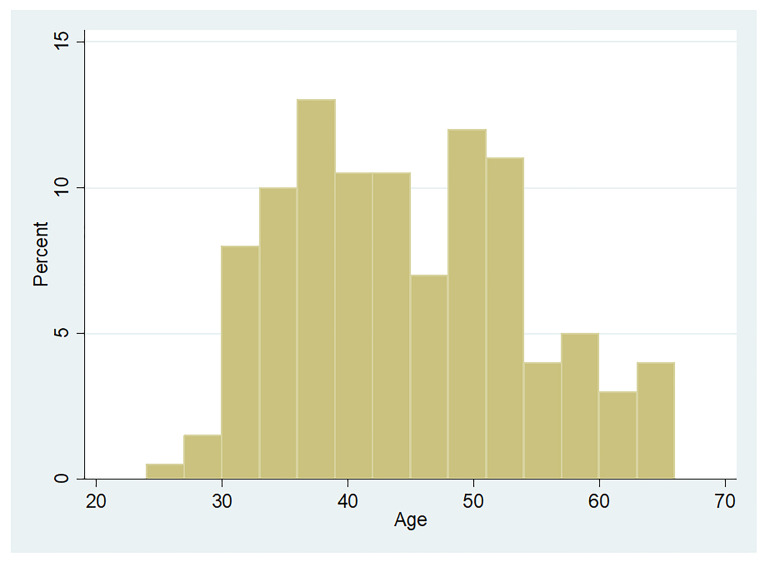
Age distribution of the Iranian drivers assessed for narcissistic symptoms.

All the drivers were male, 187(93.5%) of them being married. Mean age of the participants was 44.4 years with a standard deviation of 9.3 years. The age distribution histogram is given in Figure 1.

With respect to education level, 54% of drivers had elementary or middle school education, 77(38.5%) had high school education and 14(7%) had academic education(Table 1).

**Table 1:** The proportions of responses of the participants to the items in Narcissistic Personality Inventory-16 (NPI-16).

**I really like to be the center of attention.**	**63(32.6)**	130(76.4)	It makes me uncomfortable to be the center of attention
**I think I am a special person.**	**16(8.1)**	182(91.9)	I am no better or no worse than most people
**Everybody likes to hear my stories.**	**65(33)**	132(67)	Sometimes I tell good stories
**I insist upon getting the respect that is due me.**	**35(17.8)**	162(82.2)	I usually get the respect that I deserve
**I like having authority over people.**	**43(21.7)**	155(78.3)	I don't mind following orders
**I am going to be a great person.**	**19(9.7)**	176(90.3)	I hope I am going to be successful
**I can make anybody believe anything I want them to.**	**33(17.1)**	160(82.9)	People sometimes believe what I tell them
**I expect a great deal from other people.**	**15(7.7)**	180(92.3)	I like to do things for other people
**I like to be the center of attention.**	**35(17.9)**	161(82.1)	I prefer to blend in with the crowd
**I am an extraordinary person.**	**22(11.2)**	175(88.8)	I am much like everybody else
**I always know what I am doing.**	**131(66.5)**	66(33.5)	Sometimes I am not sure of what I am doing
**I find it easy to manipulate people.**	**6(3.1)**	189(96.9)	I don't like it when I find myself manipulating people
**People always seem to recognize my authority.**	**81(42)**	112(58)	Being an authority doesn't mean that much to me
**I know that I am good because everybody keeps telling me so.**	**117(58.8)**	82(41.2)	When people compliment me I sometimes get embarrassed
**I am apt to show off if I get the chance.**	**18(9.1)**	180(90.9)	I try not to be a show off
**I am more capable than other people.**	**7(3.5)**	193(96.5)	There is a lot that I can learn from other people

**Figure 2:**
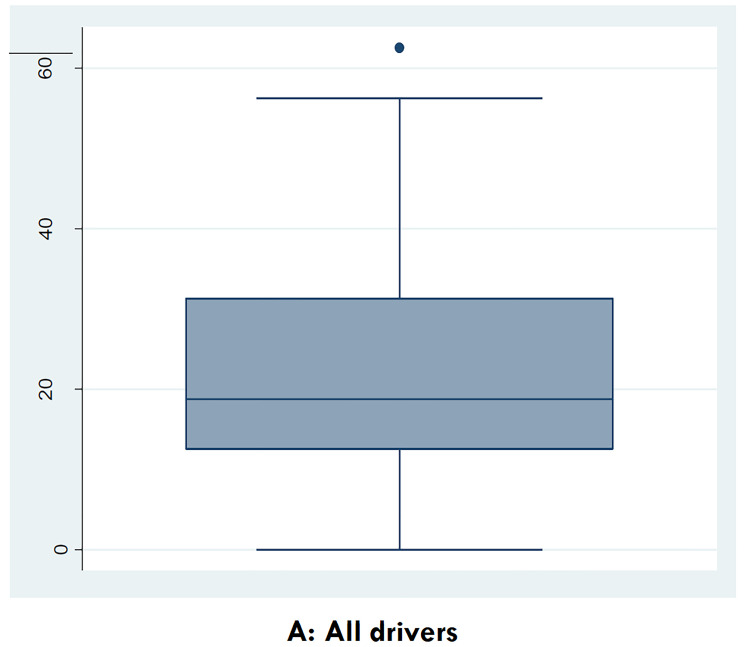

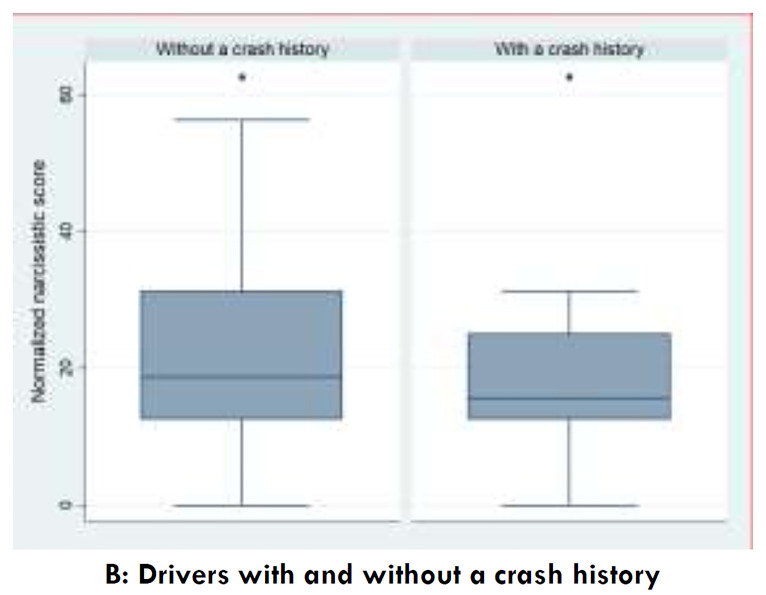

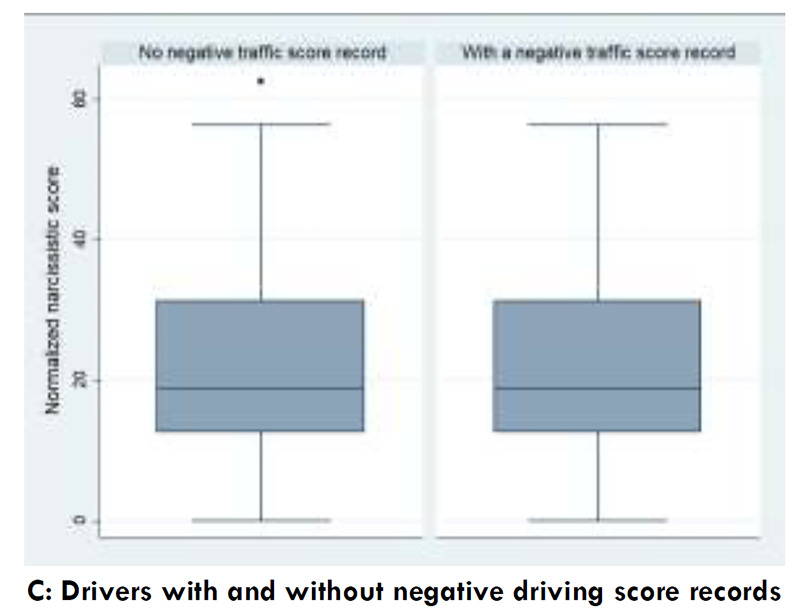
Box plots of the normalized narcissistic scores among bus drivers with respect to their crash history and negative driving score records.

The box plots of the normalized narcissistic scores among bus drivers with respect to their crash history and negative driving score records are given in Figure 2. The box lower, middle and top lines represent the 1st quartile, median and 3rd quartiles of the normalized narcissistic scores.

Fourteen drivers (7%) had a crash history over the past three years. Mean normalized narcissism score was 22.3 among those without a crash history over the past three years versus 18.8 among those with a crash history without statistical significance using independent t-test.

Forty-four drivers (22%) had a negative traffic scoring record due to high risk traffic violations registered in police database over the past three years. Mean normalized narcissism score was 22 among those without negative score record over the past three years versus 22.3 among those with a negative score history. However, the difference was not found to be statistically significant.

## Discussion

The findings of the present study did not support an association of narcissism level with risk of neither road traffic crash nor being recorded as high risk driver in police registry. A previous study was conducted on 170 undergraduate students to investigate personality and individual difference measures related to driver vengeance.^11^ These researchers used the Narcissistic Personality Inventory-40 (NPI-40) by Raskin & Terry in order to assess drivers’ level of narcissism and also the Driver Vengeance Questionnaire (DVQ) to assess driving vengeance which is considered an issue of aggressive driving and risky driving behavior. They found an association between narcissism level and driver vengeance.^11^ The difference between their study with ours should be interpreted in several ways. The first difference lies in the outcome of interest. We studied crash history and negative driving score records as outcomes, while a much complex predictors of traffic crash or extreme high risk behaviors could be expected and relationships between aggressive driving and driving outcomes are reported to be weak.^12^ Secondly, our drivers were professional drivers with a mean age of about 44 years, while the participants of mentioned study were young student drivers. The potential effect of narcissism on driving behavior may be less likely among professional drivers and those at higher age than young students. At younger age other issues such as sensation seeking and risk taking may have a more prominent role.^13-15^ A confounding effect of disorders affecting attention and impulsiveness such as adult ADHD, a known risk factor of RTIs, should also be taken into account. Adult ADHD increases the crash risk by at least 2 times and at the same time it coincides with personality disorders.^16^ Individuals with ADD, ODD and ADHD have also a tendency towards narcissism.^17^ Neither our study nor did the one by Wickens et al. have measured and controlled for a potential confounding effect of these.

Two other studies have specifically worked on narcissism in field of road safety. In one small study by Schreer in 2002, on undergraduate young population, it was shown that two narcissism subscales on the NPI-40 were significantly associated with aggressive driving behavior.^7^ A weak correlation between narcissism level and aggressive driving score was observed. Another study by Edwards et al. again on young drivers but at a larger sample of 362 drivers found that narcissism and driving anger were significant predictors of aggressive driving. They concluded, based on the theory of threatened egotism, that younger drivers with high but unstable self-esteem could react more aggressively when provoked while driving.^18^ In another study again on young students (mean age: 20), 210 drivers with different levels of narcissism were given scenarios of objectionable driving conditions requesting them to assess intentionality, inconsideration level, and anger. They were asked to indicate the behavioral responses they would likely make in similar situations. They found that drivers with higher levels of narcissistic symptoms responded more aggressively towards the frustrating driving behavior of others, but such association varied by sex and anger experience. ^8^

Long time ago, it was believed that low self-esteem, self-doubts and self-dislike could be the etiology of aggression.^8,18,19^ Nevertheless, recent evidence supports the opposite such that violence and aggression could be associated with very high, positive self-views. People having excessive unrealistic self-esteem are shown to be more likely to exhibit aggression for defending and maintaining their grandiose views.^5^ According to the above-mentioned literature, at least for the young drivers, this theory of narcissism affecting aggressive behavior could be considered in field of aggressive driving behavior but as a weak predictor of it; however, neither our results nor does the literature support a causal association between narcissism level and crash risk or being a recorded high risk driver. However, considering the complex risk profile of road traffic crashes, much larger studies are needed to rule it out.

**Limitations**

In this study some important psychiatric comorbid conditions potentially affecting driving behavior such as ADHD, borderline and antisocial personality disorders were not assessed.
